# ‘You constantly have to be switched on’: A qualitative interview study of parents of children with *STXBP1*-related disorders in the Netherlands

**DOI:** 10.1186/s13023-024-03314-7

**Published:** 2025-02-27

**Authors:** Sietske A. L. van Till, Sybren Sybesma, Hilgo Bruining, Matthijs Verhage, Eline M. Bunnik

**Affiliations:** 1 Dept. of Medical Ethics, Philosophy and History of Medicine, Erasmus MC, University Medical Centre Rotterdam, Wytemaweg 80, Rotterdam, 3015 CN The Netherlands; 2 N=You Neurodevelopmental Precision Center, Amsterdam Neuroscience, Amsterdam Reproduction and Development, Amsterdam UMC, Meibergdreef 5, Amsterdam, 1105 AZ The Netherlands; 3 Dept. of Functional Genomics Center for Neurogenomics and Cognitive Research (CNCR), VU University Amsterdam and Amsterdam UMC-Location VUmc, de Boelelaan 1085, Amsterdam, 1081 HV The Netherlands; 4 Erasmus MC, Room Na 24.19, PO box 2040, Rotterdam, 3000 CA the Netherlands

**Keywords:** Pediatrics, Caregivers, Neurodevelopmental disorders / genetics, Qualitative

## Abstract

**Background:**

Disorder-related variants in the *STXBP1* gene are increasingly detected in children with severe developmental disorders. It is commonly acknowledged that developmental disorders significantly impact family life, but little is known about the day-to-day experiences of caregivers living with children with *STXBP1*-related disorders (*STXBP1*-RD). This knowledge gap may hinder researchers and care professionals from aligning research activities, care, and support with the perspectives of parents.

**Methods:**

We conducted a semi-structured interview study to gain a better understanding of the impact of having a child with *STXBP1*-RD on daily family life. Interviews were audio-recorded, transcribed, and analyzed thematically. We developed an ‘analytical framework based on verbs’, as verbs signify action, to present our results on the experiences of parents in everyday life.

**Results:**

We conducted 16 interviews with 21 parents of children with *STXBP1*-RD (2 to 18 years old), living in the Netherlands. The respondents described their caregiving responsibilities as extremely intense and demanding. They reported being constantly occupied with caring for their child, and expressed a feeling of always being ‘switched on’. Parents’ experiences are described using the following five verbs: (1) *caring* for their child, (2) *recognizing* their child’s needs and having their child’s needs recognized, (3) *searching* for answers and suitable care, (4) *balancing* delivering care while preserving other domains of life, and (5) *coping* with emotional impacts.

**Conclusions:**

This study shows how parenting a child with *STXBP1*-RD involves continuous engagement with both child-related care responsibilities and other activities, such as arranging suitable care and coping with emotional impacts. The accumulation of these responsibilities and challenges significantly impacts the everyday lives of the entire family. To support *STXBP1* patient families, a broad approach is needed, focusing not only on developing new medical treatments, but also on improving other therapies (e.g., speech therapy or physiotherapy) and providing social support for the entire family, including emotional support, assistance with administrative tasks, and improved information provision after diagnosis.

**Supplementary Information:**

The online version contains supplementary material available at 10.1186/s13023-024-03314-7.

## Background

Currently, several hundreds of so-called monogenic neurodevelopmental disorders (mNDDs) have been described, each causing serious delays in the development of behavior and cognition in affected individuals [1]. While most scientific attention has been paid to identifying underlying pathological mechanisms and developing medical treatments, less research has been done to investigate the burden on patient families presented by these disorders. Previous research on the experiences of parents of children with neurodevelopmental disorders (NDDs) has shown that children’s disorders and the level of care they require may affect multiple domains of life, including parents’ mental wellbeing, relationships, social lives, employment status, and the lives of siblings [2–5]. More insight into these challenges and how they are experienced by families of children with specific genetic neurodevelopmental disorders is directly needed.

In this context, *STXBP1*-related disorders (*STXBP1*-RD) are a group of rare mNDDs characterized by severe developmental delays and a broad array of neurological, psychiatric, and somatic symptoms [1, 6, 7]. This group of disorders, identified in 2008, is caused by *de novo* mutations in or deletions of the *STXBP1* gene [8]. Available research has indicated that almost all children diagnosed with *STXBP1*-RD have an intellectual disability and developmental delay, and 89% are affected by epilepsy/seizures [6, 7]. Clinical features of *STXBP1*-RD include autism, speech problems, challenging behavior, gastrointestinal problems, and motor impairments often resulting in wheelchair dependence. The clinical presentation varies widely within the *STXBP1* patient group ranging from mild to very severe [9]. Children with *STXBP1*-RD are assumed to require continuous care and support from caregivers throughout the day [10]. *STXBP1* patient families frequently go through a long and burdensome diagnostic odyssey due to children’s complex combinations of symptoms that may not be immediately recognizable to healthcare professionals [9, 11]. Even after diagnosis, parents are often left with insufficient information regarding the child’s prognosis, and no disease-modifying treatments exist [10, 12].

Fifteen years ago, *STXBP1*-RD was not classified as a distinct group of disorders, resulting in zero identified patients. To date, over 500 cases have been reported. As the estimated prevalence of *STXBP1*-RD is 1 in 30,000 individuals, the number of *STXBP1* diagnoses is expected to increase, particularly among children with early-onset epilepsy [1, 7, 13]. The extensive framework of knowledge available for other NDDs is still completely lacking for this group, and needs to be developed. To gain a comprehensive understanding of *STXBP1*-RD, and ultimately to improve care for children with *STXBP1*-RD and their families, various research groups are focusing on this patient population, including the *European STXBP1 Consortium* (ESCO), the *STXBP1 Clinical Trial Ready* (STARR) study in the US, and the Dutch BRAINmodel project [14–16]. In recent years, significant progress has been made in understanding the etiology and clinical symptoms. This knowledge now serves as the foundation for research projects aimed at developing *medical* therapies, including gene therapies and antisense therapies [12]. However, it is still unclear whether this focus aligns with the needs and preferences of patients and their caregivers. To improve the relevance of research, it is crucial to involve patient communities, particularly in shaping research agendas and research objectives. However, initial discussions with parents of children with mNDDs participating in the BRAINmodel users-committee revealed a lack of research on the needs and preferences of children with *STXBP1*-RD and their caregivers.

Limited qualitative research exists that focuses on the experiences of parents of children with specific mNDDs, such as *STXBP1*-RD. So far, one qualitative study has been conducted that exclusively investigated the experiences of parents and caregivers of children with *STXBP1*-RD (i.e., 19 caregivers and 7 healthcare professionals) [17]. This study showed that developmental delays, behavioral symptoms, and seizures were the most frequently reported symptoms by caregivers of children with *STXBP1*-RD. The most mentioned impacts of the disorder on caregivers were the emotional impacts, effects on families’ support systems, and required changes to daily life and activities. While this study showed that there are impacts of the child’s disorder on caregivers in several domains of everyday life, it did not describe what these impacts are. Hence, the actual impact – emotionally, socially, and practically – of living with a child with *STXBP1*-RD, from the perspective of parents, remains unclear. Consequently, it remains challenging for researchers, to align priorities in mNDD research with parental perspectives.

We have undertaken an interview study to capture parents’ experiences of their day-to-day life with their children with *STXBP1*-RD. With this interview study, we aspire to uncover *what the impact is* of being a parent of a child with *STXBP1*-RD. A better understanding thereof will help determine how the needs of families of children with mNDDs can be met and how meaningful advancements in care and support for *STXBP1* patient families can be achieved.

## Methods

This interview study was conducted within the context of the BRAINmodel project: an interdisciplinary consortium which aims to investigate the pathophysiology of various rare mNDDs, such as *STXBP1*-RD, and to ultimately discover therapeutic targets for the development of personalized treatments using stem cell-based neuronal cell models [16]. We conducted a semi-structured interview study to investigate the experiences of parents of children with *STXBP1*-RD and their perspectives on new research initiatives, using an interview guide [Table 1, Appendix 1 for complete version]. The interview guide was developed in collaboration with medical professionals and representatives of patient organizations (ZeldSamen, IDefine, and the *STXBP1* Foundation). For the design of our study, we used the *consolidated criteria for reporting qualitative research* (COREQ) checklist [18].

**Table 1:** Abbreviated interview guide

Main questions: - How did you experience the diagnostic process? - What did the diagnosis mean to you and your family? - What symptoms does your child have, and what is the (most important) impact of these symptoms on daily life? - How does caring for your child affect your family (emotionally, socially, practically)? - What support, care, and treatments is your child/family currently receiving? To what extent do these meet your family’s needs? - What *are* your family’s *needs*? - What are your perspectives on and expectations of new research initiatives (like the BRAINmodel project) and the development of new medical treatments?	

### Participants

The participants were recruited by purposive sampling, including snowball sampling. The inclusion criteria were: (a) being a parent of a child with a *STXBP1* gene mutation/deletion up to 18 years of age, and (b) being fluent in Dutch. Parents living across the Netherlands who consented to being approached for research participation were invited by a clinical geneticist affiliated with the AmsterdamUMC (MM) either via email (*N* = 14) or via phone call (*N* = 2). A total of 16 parents of children with *STXBP1*-RD were invited, of whom 10 participated in the study. One parent was referred by a researcher at the Erasmus Medical Centre (RL) to the BRAINmodel research group. Additionally, the interview study was announced in two newsletters: ZeldSamen, a magazine of the Dutch patient organization for parents of children with rare NDDs, and the *STXBP1* newsletter provided by the N = You Neurodevelopmental Precision Center. The researchers’ contact details were provided in these announcements. Six parents contacted researchers from the BRAINmodel research group (ST, AB, TK), of whom five parents were interviewed. Parents who did not respond to the invitations did not provide reasons for opting not to participate.

### Interviews

ST conducted all interviews between June 2022 and April 2023, either face-to-face at respondents’ homes (*N* = 12) or by video call (*N* = 4), based on respondents’ preferences. The participants could choose to be interviewed alone or as a parent couple. The interviews lasted between 74 and 141 min. The participants provided verbal informed consent for study participation, which was recorded on audio-tape. Interviews were audio-recorded and transcribed *verbatim* by Transcriptie Online, a professional transcription-assistance company in the Netherlands. The transcripts were anonymized. Data were stored and handled in accordance with the General Data Protection Regulation and the Privacy Regulations of Erasmus MC. For this study, we received a waiver from the research ethics review committee of Erasmus MC, as the committee assessed that this type of study does not fall under the scope of the Medical Research Involving Human Subjects Research Act (WMO).

### Data analysis

Two researchers (ST and SS) independently coded the data using NVivo software (version 12 QRS-international). A codebook was drafted based on the coding of the first three transcripts using open coding, and was iteratively revised until data saturation was reached, i.e., when no additional codes were identified in the transcripts. The coded data were analyzed independently by two researchers (ST and SS) using thematic analysis. Themes were derived from coded data that described the experiences of parents of children with *STXBP1*-RD [19]. Discrepancies in the interpretation of the data were solved through discussion with a third researcher (EB). While the interviews were discussed, researchers observed that parents often spoke about their experiences in active terms, spontaneously using similar verbs. In the final stage of the analysis, ST, SS, and EB labeled the identified themes as verbs, because verbs signify ‘action’. We used this approach to organize identified themes into a coherent account, which we named an ‘analytical framework based on verbs’. This framework represents parents’ experiences of always being ‘occupied’ or ‘switched on’, and assists in providing a better understanding of these experiences.

## Results

### Sample characteristics

Sixteen interviews were conducted with 21 parents of 16 children with *STXBP1*-RD [Appendix 2]. Nine mothers, two fathers, and five couples of parents were included. Thirteen parents were in a relationship/married. Five parents had a full-time job, 12 had a part-time job, and four were unemployed (temporarily or permanently).

The children were 2 to 18 years old. Five children were 2 to 6 years old, seven children were 7 to 11 years old, and four children were 12 to 18 years old. There were 10 boys and six girls. The children had different clinical phenotypes as described by their parents. All children, except for one, had an intellectual disability, ranging from mild to severe. Six children had epilepsy, and six others had had epilepsy in the past, of whom three were still using antiepileptic drugs. All children had developmental delays. At the time of the interviews, five children were verbal and 11 children were non-verbal, and 12 out of 16 children could walk without assistance.

### Themes

We identified the following five themes that accumulated in parents’ day-to-day experiences of being constantly ‘switched on’: (1) caring, (2) recognizing, (3) searching, (4) balancing, and (5) coping [Table 2]. Respondents stated that having a child with *STXBP1*-RD means that they are constantly occupied with their child and with care responsibilities. We developed an ‘analytical framework based on verbs’ to present five themes that characterize the activated state described by the parents.

**Table 2:**
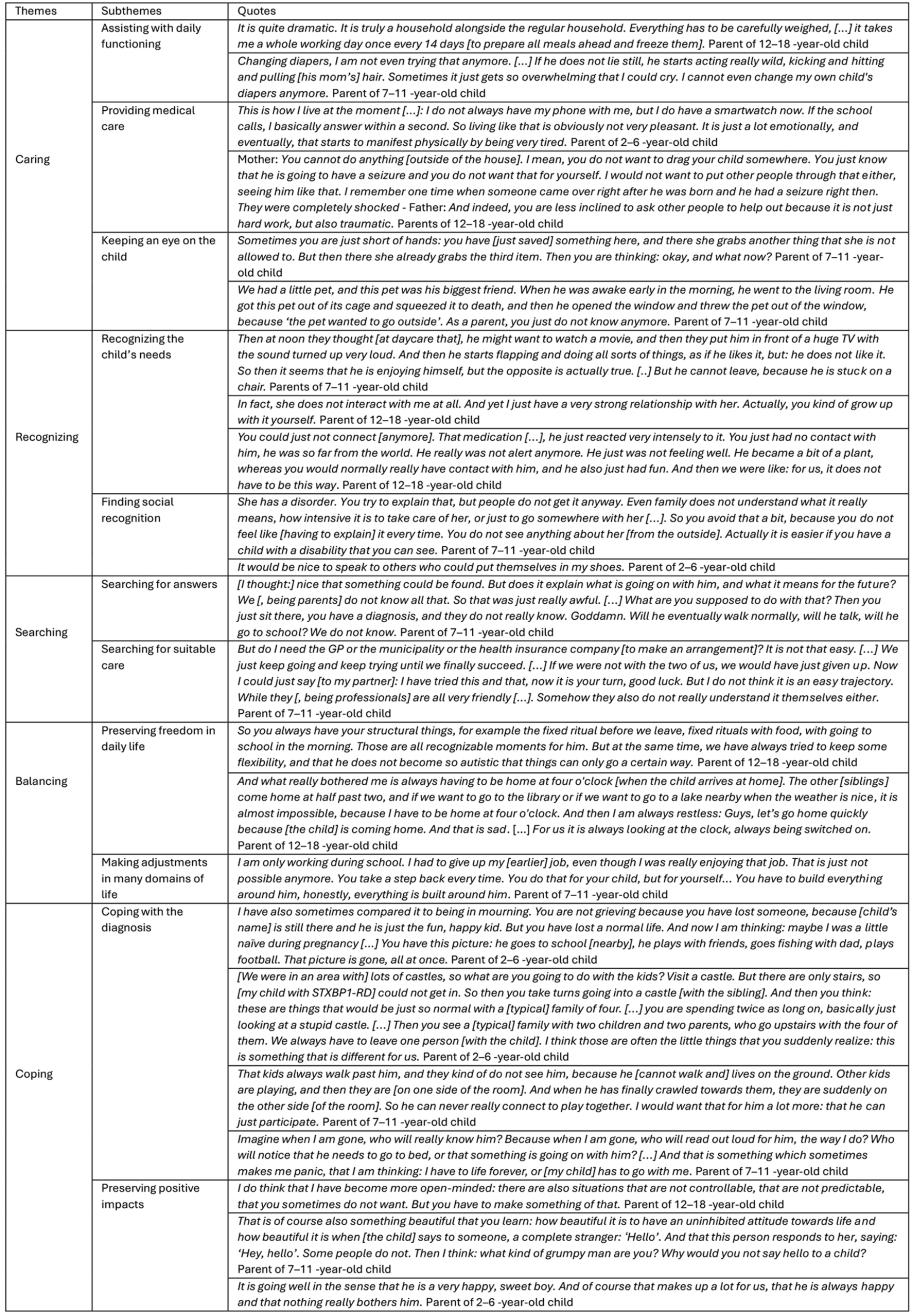
Themes (verbs) and quotes describing the experiences of parents of children with STXBP1-RD

**1. Caring**

The respondents described how they had to secure the safety of the child and care for all the child’s basic and medical needs.

#### Assisting with daily functioning

The respondents described how they assisted their child with daily functioning, and tended to its basic needs and physical and mental wellbeing. Parental engagement in providing day-to-day care varied with the child’s developmental stage and clinical symptoms. Some children needed assistance with eating. One parent illustrated how he was occupied with his child’s medical diet:

*It is quite dramatic. It is truly a household alongside the regular household. Everything has to be carefully weighed*, *[…] it takes me a whole working day once every 14 days [to prepare all meals ahead and freeze them].* Parent of 12–18 -year-old child.

Respondents mentioned the impact of their child’s motoric problems, leading, for instance, to wheelchair dependence or incontinence. These symptoms required parents to lift their child and change diapers, which placed substantial physical demands on them. The respondents often noted that the burden of this care increased as the child grew older, bigger, and stronger:

*Changing diapers*, *I am not even trying that anymore. […] If he does not lie still*, *he starts acting really wild*, *kicking and hitting and pulling [his mom’s] hair. Sometimes it just gets so overwhelming that I could cry. I cannot even change my own child’s diapers anymore.* Parent of 7–11 -year-old child.

#### Providing medical care

The respondents explained that they had to take care of their child’s medical problems, among which epilepsy was most pronounced. Several children suffered from epilepsy of varying severity. A few children had numerous seizures per day. The respondents described significant emotional and practical impacts, such as challenges in finding effective medical treatments, feelings of powerlessness, and increased dependency of their child during periods of frequent seizures:

Mother: *You cannot do anything [outside of the house]. I mean*, *you do not want to drag your child somewhere. You just know that he is going to have a seizure and you do not want that for yourself. I would not want to put other people through that either*, *seeing him like that. I remember one time when someone came over right after he was born and he had a seizure right then. They were completely shocked -* Father: *And indeed*, *you are less inclined to ask other people to help out because it is not just hard work*, *but also traumatic.* Parents of 12–18 -year-old child.

Even if the child did not had epilepsy, respondents were often aware of the elevated prevalence of epilepsy within the *STXBP1* patient group, and remained watchful for early indicators. A mother expressed fear that her child might develop epilepsy, expecting emergency calls from school:

*This is how I live at the moment […]: I do not always have my phone with me*, *but I do have a smartwatch now. If the school calls*, *I basically answer within a second. So living like that is obviously not very pleasant. It is just a lot emotionally*, *and eventually*, *that starts to manifest physically by being very tired.* Parent of 2–6 -year-old child.

#### Keeping an eye on the child

*Caring* also entails ensuring the safety of the child and its surroundings. Cognitive impairments rendered the children unable to assess dangerous situations, demanding continuous parental supervision. Some children expressed exploratory behavior and sought external stimuli. Some respondents described how children damaged objects or harmed themselves or others, including their caregivers or siblings, by biting, hitting, or pulling hair. These respondents emphasized that their child had no bad intentions but instead simply did not understand the consequences of its actions. The parents constantly had to keep an eye on their child and remain ready to intervene:

*Sometimes you are just short of hands: you have [just saved] something here*, *and there she grabs another thing that she is not allowed to. But then there she already grabs the third item. Then you are thinking: okay*, *and what now?* Parent of 7–11 -year-old child.

Some respondents said that they had to be in the same room as their child 24/7, and could not quickly visit the bathroom. Physical safety was a bigger concern when the child could walk independently. Several respondents described situations in which they could not control their child’s behavior. A mother recalled:

*We had a little pet*, *and this pet was his biggest friend. When he was awake early in the morning*, *he went to the living room. He got this pet out of its cage and squeezed it to death*, *and then he opened the window and threw the pet out of the window*, *because ‘the pet wanted to go outside’. As a parent*, *you just do not know anymore.* Parent of 7–11 -year-old child.

**2. Recognizing**

The parents often used the verb ‘recognizing’ when they described their experiences and relations with others. They attributed two different meanings to ‘recognizing’. First, the parents had to be constantly alert to recognize their child’s needs. Second, the parents mentioned *seeking recognition* for their family’s needs by those in their social network.

#### Recognizing the child’s needs

The respondents mentioned that they were constantly alert and engaged with trying to recognize their child’s needs. Many children had speech problems that limited their ability to verbalize their needs and emotions. These children interacted with their family members in various ways, including sounds and behaviors. The respondents felt a responsibility to be present to protect their child’s interests because they, and sometimes the child’s siblings, were the only ones capable of ‘reading’ the child and noticing signs that suggest something is going on. Yet other respondents also indicated challenges in interpreting the child’s ‘little signs’. A couple narrated how their child was suffering from sleeping problems. The child appeared to be overstimulated during school days, which had not been recognized by the teachers:

*Then at noon they thought [at daycare that]*, *he might want to watch a movie*, *and then they put him in front of a huge TV with the sound turned up very loud. And then he starts flapping and doing all sorts of things*, *as if he likes it*, *but: he does not like it. So then it seems that he is enjoying himself*, *but the opposite is actually true. […] But he cannot leave*, *because he is stuck on a chair.* Parents of 7–11 -year-old child.

Even if the children could not verbally communicate, the respondents described close relationships with them:

*In fact*, *she does not interact with me at all. And yet I just have a very strong relationship with her. Actually*, *you kind of grow up with it yourself.* Parent of 12–18 -year-old child.

For some respondents, the interaction with their child was an important consideration when deciding on the use of medication. A few respondents had experienced that antiepileptic drugs had diminished their child’s expressive abilities:

*You could just not connect [anymore]. That medication […]*, *he just reacted very intensely to it. You just had no contact with him*, *he was so far from the world. He really was not alert anymore. He just was not feeling well. He became a bit of a plant*, *whereas you would normally really have contact with him*, *and he also just had fun. And then we were like: for us*, *it does not have to be this way*. Parent of 12–18 -year-old child.

In addition, some parents had to adapt their ways of interacting with their child as their child’s expressive abilities evolved over time, for example when their child’s development had deteriorated due to prolonged epilepsy.

#### Finding social recognition

The parents sought recognition within their social network. The respondents sometimes encountered misunderstandings within their close social circle, including family and friends, as well as among ‘outsiders’, or professionals at administrative institutions. Others did not always understand that caring for a child with a rare disorder requires an extreme investment of energy and organizational skills:

*She has a disorder. You try to explain that*, *but people do not get it anyway. Even family does not understand what it really means*, *how intensive it is to take care of her*, *or just to go somewhere with her […]. So you avoid that a bit*, *because you do not feel like [having to explain] it every time. You do not see anything about her [from the outside]. Actually it is easier if you have a child with a disability that you can see.* Parent of 7–11 -year-old child.

Sometimes the *STXBP1*-diagnosis brought recognition of the child’s special needs. It helped parents to explain the child’s disorder to others and validated their need for care and support.

Finally, many respondents aspired to find recognition in other parents, seeking to share experiences and find support. Peer support allowed some parents to recognize traits of their child in other children with *STXBP1*-RD. Some respondents would have appreciated (more) interaction with other parents: *“It would be nice to speak to others who could put themselves in my shoes.”* (Parent of 2–6 -year-old child). Social media groups and *STXBP1* family meetings were highly valued by some parents. Nevertheless, finding recognition was described as challenging by most respondents due to the rarity of the disorder and, also, the heterogeneity of clinical phenotypes: every family has its own personal experiences. A mother said that despite the existence of an *STXBP1*-community, she felt like she “*stands alone”*.

**3. Searching**

The respondents indicated that a significant proportion of their efforts was devoted to searching for answers about their child’s disorder, and for suitable care.

#### Searching for answers

The respondents had been through long diagnostic odysseys before the *STXBP1*-diagnosis was ultimately confirmed, and thereafter had to persist in searching for answers. Often, at diagnosis, healthcare professionals could only provide limited information about *STXBP1*-RD. Parents often consulted the internet, but again had found only limited information. Those parents continued to experience feelings of uncertainty due to the heterogeneity of *STXBP1* clinical phenotypes. Parents had questions about how the disorder would develop in their child, which strategies were most promising to foster its development, and whether there were new therapeutic approaches that would benefit the child:

*[I thought: ] nice that something could be found. But does it explain what is going on with him*, *and what it means for the future? We [*, *being parents] do not know all that. So that was just really awful. [...] What are you supposed to do with that? Then you just sit there*, *you have a diagnosis*, *and they do not really know. Goddamn. Will he eventually walk normally*, *will he talk*, *will he go to school? We do not know.* Parent of 7–11 -year-old child.

A few respondents characterized the *STXBP1*-diagnosis as inconsequential, due to the unavailability of information, appropriate care, and treatment options.

#### Searching for suitable care

The respondents had to arrange care for their child, including searching for suitable care and support services, setting up contracts with care providers, and navigating through administrative procedures for access and reimbursement. They reported having to adjust their homes, and arrange overnight care facilities on weekends, medical equipment (e.g., wheelchairs), therapies (e.g., speech- or physiotherapy), sports facilities, and individual assistance at home. The parents expressed significant uncertainty about the availability of care for children with *STXBP1*-RD. They mentioned various barriers, such as long waiting lists, differences in administrative processes between municipalities, and a lack of knowledge and expertise among care providers. Also, the respondents noted the complexity of dealing with a multitude of care providers and administrative institutions, highlighting insufficient coordination and harmonization among them:

*But do I need the GP or the municipality or the health insurance company [to make an arrangement]? It is not that easy. […] We just keep going and keep trying until we finally succeed. […] If we were not with the two of us*, *we would have just given up. Now I could just say [to my partner]: I have tried this and that*, *now it is your turn*, *good luck. But I do not think it is an easy trajectory. While they [*, *being professionals] are all very friendly […]. Somehow they also do not really understand it themselves either.* Parent of 7–11 -year-old child.

Encountering barriers had significant emotional impacts, including frustration and uncertainty. A father said that due to uncertainties around care arrangement, *“it seems you are also a bit at the gods’ mercy.”* A few respondents had concerns about equal access to care: some parents might be more knowledgeable, assertive, or strategic in navigating through administrative procedures than others.

Furthermore, parents could not always find full financial compensation. Some respondents indicated that the ‘personal budget’ assigned to their child was insufficient to cover (all) financial costs. Some health or social care facilities were expensive, forcing parents to prioritize. Physiotherapy or vans suitable for transporting wheelchairs were not always reimbursed, while they were often very expensive. Seemingly minor out-of-pocket expenses could also add up to large sums. For instance, some respondents had to buy new clothes on a weekly basis because of their child’s tendency to demolish things.

**4. Balancing**

The respondents described life as a complicated act of balancing caring for their child, while preserving the functioning of their families in other domains of life. They experienced their family situation as ‘not normal’, and frequently had to make significant adjustments or sacrifices to integrate child-related activities (caring, recognizing, searching) into their daily lives.

#### Preserving freedom in daily life

Some respondents highlighted limited freedom and flexibility in everyday activities as the most significant impact on the family. The parents faced constraints in daily activities, for instance when their child was unable to join outdoor activities and had to stay at home, supervised. The respondents described trying to organize their lives around their child’s needs while also trying to strengthen their child’s resilience. A couple explained how they encouraged their child to deal with unexpected situations:

*So you always have your structural things*, *for example the fixed ritual before we leave*, *fixed rituals with food*, *with going to school in the morning. Those are all recognizable moments for him. But at the same time*, *we have always tried to keep some flexibility*, *and that he does not become so autistic that things can only go a certain way.* Parents of 12–18 -year-old child.

Some respondents worried that the child’s siblings did not experience the freedom they otherwise would have enjoyed, particularly on weekends and holidays. The parents strove to balance caring for their child with *STXBP1*-RD while ensuring that siblings received sufficient care and attention from their parents.

*And what really bothered me is always having to be home at four o’clock [when the child arrives at home]. The other [siblings] come home at half past two*, *and if we want to go to the library or if we want to go to a lake nearby when the weather is nice*, *it is almost impossible*, *because I have to be home at four o’clock. And then I am always restless: Guys*, *let’s go home quickly because [the child] is coming home. And that is sad*. […] *For us it is always looking at the clock*, *always being switched on.* Parent of 12–18 -year-old child.

#### Making adjustments in many domains of life

Respondents felt restricted in their personal lives when having to adjust or reduce their work, or feeling reluctant to engage in social activities:

*I am only working during school. I had to give up my [earlier] job*, *even though I was really enjoying that job. That is just not possible anymore. You take a step back every time. You do that for your child*, *but for yourself… You have to build everything around him*, *honestly*, *everything is built around him.* Parent of 7–11 -year-old child.

The respondents touched upon various domains of life in which adaptations were necessary to accommodate their child’s needs. Respondents mentioned impacts on relationships. One respondent narrated how their child’s constant need for supervision implied that he and his partner led largely separate lives: they could almost never undertake activities together. Other respondents mentioned having different ways of providing care or coping with emotional impacts then their partners. Furthermore, a few mothers noted that their child’s disorder influenced their family planning. These mothers had decided against having another child, because they feared additional burdens on their family. Moreover, some respondents had physical problems, such as back or shoulder problems, resulting from repetitive movements associated with caregiving. Physical manifestations of emotional stress, such as extreme fatigue, were mentioned by respondents as well. These respondents tried to balance the physical load of caring for their child against getting sufficient rest to stay healthy. In some families, balance could only be achieved when their child (at least partly) lived in an assisted living facility.

**5. Coping**

Besides the practical and visible challenges, all parents reported that they had to cope with the emotional impacts of their child’s disorder.

#### Coping with the diagnosis

Some respondents indicated that – though unsettling at first – they had become habituated to their situation, and that the genetic diagnosis had helped. It provided mental peace and validation of their concerns. It proved that they were ‘*not crazy*’ nor culpable, and that the cause of the disorder was not hereditary. Yet most respondents acknowledged the severe emotional impact of the diagnosis, accompanied by feelings of sorrow and hopelessness:

*I have also sometimes compared it to being in mourning. You are not grieving because you have lost someone*, *because [child’s name] is still there and he is just the fun*, *happy kid. But you have lost a normal life. And now I am thinking: maybe I was a little naïve during pregnancy […] You have this picture: he goes to school [nearby]*, *he plays with friends*, *goes fishing with dad*, *plays football. That picture is gone*, *all at once.* Parent of 2–6 -year-old child.

For some respondents the emotional impact was most pronounced immediately after the diagnosis, whereas for others, coping with the diagnosis rather entailed a prolonged and ongoing process. The respondents elaborated on the emotional impact of the ‘little things’: seemingly small moments in everyday life that confronted parents with their child’s disorder. To illustrate, a mother talked about a holiday experience in an area with:

… *lots of castles*, *so what are you going to do with the kids? Visit a castle. But there are only stairs*, *so [my child with STXBP1-RD] could not get in. So then you take turns going into a castle [with the sibling]. And then you think: these are things that would be just so normal with a [typical] family of four. […] you are spending twice as long on*, *basically just looking at a stupid castle. […] Then you see a [typical] family with two children and two parents*, *who go upstairs with the four of them. We always have to leave one person [with the child]. I think those are often the little things that you suddenly realize: this is something that is different for us.* Parent of 2–6 -year-old-child.

Also, confrontations with the child’s impairments were emotionally burdensome. A mother explained how it was painful to see her child’s limited abilities to connect with others:

*That kids always walk past him*, *and they kind of do not see him*, *because he [cannot walk and] lives on the ground. Other kids are playing*, *and then they are [on one side of the room]. And when he has finally crawled towards them*, *they are suddenly on the other side [of the room]. So he can never really connect to play together. I would want that for him a lot more: that he can just participate.* Parent of 7–11 -year-old child.

Furthermore, the respondents coped with concerns about their child’s future, for instance, about the implications of puberty, the possibility that their child might develop epilepsy in the future, or that existing epilepsy might worsen. They expressed concerns about the future, when they may no longer be capable of caring for their child. For some respondents, the prospect of their child outliving them was frightening.

*Imagine when I am gone*, *who will really know him? Because when I am gone*, *who will read out loud for him*, *the way I do? Who will notice that he needs to go to bed*, *or that something is going on with him? […] And that is something which sometimes makes me panic*, *that I am thinking: I have to life forever*, *or [my child] has to go with me.* Parent of 7–11 -year-old child.

The respondents indicated that emotional support was often not offered during and following the diagnostic process.

#### Preserving positive impacts

Lastly, the respondents mentioned positive impacts of their child’s disorder on the family. They made more conscious choices throughout their lifetime, and had become more flexible and more compassionate towards the private situations of others:

*I do think that I have become more open-minded: there are also situations that are not controllable*, *that are not predictable*, *that you sometimes do not want. But you have to make something of that.* Parent of 12–18 -year-old child.

Moreover, parental descriptions of the child’s personal characteristics often had a positive connotation. Children were characterized as being humorous, cheerful, and happy. According to respondents, children enjoyed social interactions with others:

*That is of course also something beautiful that you learn: how beautiful it is to have an uninhibited attitude towards life and how beautiful it is when [the child] says to someone*, *a complete stranger: ‘Hello’. And that this person responds to her*, *saying: ‘Hey*, *hello’. Some people do not. Then I think: what kind of grumpy man are you? Why would you not say hello to a child?* Parent of 7–11 -year-old child.

The respondents described how their children found joy in activities that were in accordance with their capacities, such as listening to music, or jumping on a trampoline. Some respondents noted that their child generally seemed quite content, and that, for them, prioritizing their child’s wellbeing was the most important strategy for coping with their child’s disorder:

*It is going well in the sense that he is a very happy*, *sweet boy. And of course that makes up a lot for us*, *that he is always happy and that nothing really bothers him.* Parent of 2–6 -year-old child.

## Discussion

This study provides a comprehensive overview of the experiences of parents of children with *STXBP1*-RD, using an ‘analytical framework based on verbs’ to underline the ‘actions’ parents have to undertake in everyday life when caring for their children. Our results illustrate that these parents are constantly occupied with both caring for their child and other activities, such as arranging suitable care and finding recognition in their social environment. Most families face incredible challenges to organize care and support to cope with the burden presented by *STXBP1*-RD. This study shows how parental experiences are shaped not only by the challenges faced by the parents, but also by the family’s resilience in coping with them. Often parents have to change their employment status and families experience limited flexibility in social activities. Moreover, parents must cope with emotional impacts, as they are persistently confronted with their child’s disorder, constraints in daily life, and concerns about the future.

Our findings align with earlier empirical research on families with children with NDDs and rare genetic disorders that has shown that these disorders and caregiving responsibilities have adverse impacts on the mental and physical wellbeing of parents and parents’ social activities [3, 4, 20]. Spindler et al. have shown that among parents of children with seizure disorders and developmental disorders, 51% changed their employment status, 20% changed their habitation, and 15% broke up [2]. Sullivan et al. have shown that the most mentioned impacts of the disorder on caregivers of children with *STXBP1*-RD living in the US were emotional impacts, support systems, and impacts on freedom and flexibility in daily life [17]. Our study confirms the presence of these impacts on various domains of life and contributes to a deeper understanding of these impacts. First, emotional impacts are not limited to the initial diagnosis phase but also continue to affect parents throughout their lives. Second, it is not the child’s neurological, somatic, or behavioral symptoms, per se, nor in isolation, that explain the significant impact on the daily lives of families living with children with *STXBP1*-RD, but rather their accumulation, and the resulting complexity and extent of care and support required to preserve their child’s wellbeing. Taken together, this places substantial emotional, practical, and physical demands on parents. This study thus *documents*, and helps explain, parental perspectives on previously reported impacts.

Our findings have implications for: (1) the impact of an *STXBP1*-diagnosis, (2) aligning medical and social support with parental needs and preferences, (3) the concept of personal identity in children with NDDs (Table 3).

**Table 3:** Summary of the key findings and main implications of this study

Key findings:
- Parents are occupied with both child-related care responsibilities and other activities, such as arranging suitable care, maintaining the family’s functioning in various other domains of daily life, and coping with emotional impacts. - STXBP1 patient families face incredible challenges to organize care and support to cope with the burden presented by STXBP1-RD.
**Main implications:**
- To support *STXBP1* patient families, a broad approach is needed, encompassing the availability of both medical care and psychosocial interventions for the family as a whole. - To support parents during and after the diagnostic process, emotional support should be offered and information provision needs to be improved. - Simplifying and standardizing administrative processes, and providing professional assistance and better information are essential steps to improve access to necessary care and support for all *STXBP1* families. - Parents support the development of new medical treatments. Still, (new) *medical* therapies should not be pursued at all costs: children’s positive characteristics should be acknowledged and preserved.

First, our study serves as an exploratory case to gain better insight into the experiences of mNDD patient families. Rare genetic syndromes like *STXBP1*-RD are better and more quickly diagnosed in children. Genetic diagnoses have both positive and negative implications. On the one hand, a genetic diagnosis can contribute to relief and acceptance, and can enable parents to connect with peers [21, 22]. Furthermore, it can improve social recognition and access to medical and social support [23]. On the other hand, parents experience loss and sorrow when their child appears to have a chronic disorder [21, 24]. Moreover, uncertainty persists when genetic diagnoses are associated with broad spectra of clinical presentations [23, 25].

Likewise, our interview study shows varied parental experiences with the attribution of the *STXBP1*-diagnosis. Parents appreciated the practical benefits of their children having a ‘label’ and its contribution to acceptance and mental peace. Still, these parents were devastated and felt they lacked standardized treatment plans, emotional support, comprehensive information, and assistance in arranging appropriate care. The heterogeneity in clinical presentation within the *STXBP1* patient group led to uncertainty among parents regarding their child’s prognosis, and to challenges in finding recognition through peer support. The parents provided valuable suggestions for healthcare professionals to improve support to parents during and after the diagnostic process. They emphasized the need for emotional support, which is currently not offered often in the Dutch healthcare system, and comprehensive information covering the full range of *STXBP1* clinical phenotypes, including both mild and severe phenotypes. Lastly, parents supported research projects that investigate *STXBP1* disease mechanisms to promote the understanding of this disorder.

Second, to improve care and support for patient families, a comprehensive understanding of parental needs and preferences is required. In accordance with the study by Sullivan et al., parents in our study described their children’s epilepsy, behavioral symptoms, speech problems, motoric impairments, and cognitive impairments as most pronounced [17]. Research efforts often focus on identifying medical treatments to reduce clinical symptoms. Although some parents supported potential medical treatments (e.g., to control the child’s epilepsy or to improve motoric or cognitive development), other parents expressed more need for other therapies, such as physiotherapy to improve the child’s mobility (which is sometimes not reimbursed by Dutch health insurers) and speech therapies. Moreover, we show how external factors impact parental experiences, such as lack of understanding within the family’s social network and barriers in accessing appropriate care and support. To alleviate the burden that is put on a family and to increase the family’s resilience, parents not only need research efforts within the medical domain, but parents rather need interventions within the social domain.

Specifically, parents highlighted the burdens of their role as care coordinator. In the Dutch healthcare system, parents not only provide primary care for their children but also act as care coordinators. Parents are responsible for purchasing care (including support at home, transport, or day care), and often need indications for this care, provided by the municipality [26]. Parents need to identify local care providers and set up contracts with them. The administrative workload is time-consuming and emotionally stressful for these parents. Due to complex administrative processes, some parents (e.g., those who are highly educated and/or have larger social networks) might be more successful in arranging care than others, which heightens the risk of exacerbating socioeconomic and demographic inequality. Yet, this issue is not unique to *STXBP1* patient families in the Netherlands. It is also observed in other patient families worldwide [20, 27–29]. Efforts to support patient families should encompass not only developing medical treatments, but also strengthening social systems to increase the accessibility of care and support. Simplifying and standardizing administrative processes, and providing professional assistance and better information are essential steps to improve access to necessary care and support for all *STXBP1* families.

Third, this study has implications for defining the concept of personal identity in children with NDDs. The parents in our study often described in positive terms their child’s personal character (e.g., being cheerful) and behavior (e.g., making jokes and non-verbal interactions). These parental perspectives align with the published literature, describing children with *STXBP1*-RD as individuals with remarkable social tendencies [30]. While most parents desired improvements in their child’s condition, *medical* therapies should, according to parents, not be pursued at all costs. In balancing family life with the child’s required care, the positive characteristics of the child should be acknowledged and preserved. Turbitt et al. referred to empirical research on the perspectives of parents of children with NDDs (e.g., with Down syndrome) to show that for some parents, the impact of new treatments or ‘cures’ on children’s personal identity and authenticity might be a concern [31, 32]. In our study, a few parents mentioned the negative impacts of antiepileptic drugs on their child’s wellbeing and expressive abilities: the treatments *changed* their child. Yet, as epilepsy can adversely impact the child’s development and wellbeing, it was seen by the same parents as a potential threat to the child. Also, some parents felt that the child’s developmental delays should have been (medically) treated as it hinders the child’s self-determination and future capacities. As parents were concerned about personal identity and authenticity in the context of developing medical treatments for children with *STXBP1*-RD, yet the meanings of these concepts are unclear, further conceptual research is needed. Parents’ perspectives provide valuable insights into the ethical considerations in developing medical treatments for children with *STXBP1*-RD. This study thus demonstrates the importance of investigating parental perspectives to inform researchers in guiding research efforts in meaningful ways.

### Limitations

The first limitation of this study is that only parents living in the Netherlands were interviewed. Parental experiences may have been influenced by external factors such as existing local healthcare systems and socio-cultural contexts. Nevertheless, the identified themes align with those found in the international scientific literature. Therefore, we believe that our results contribute to a better understanding of the impact of the child’s disorder and care responsibilities on *STXBP1* patient families on an international scale. Further empirical research on experiences of parents with children with *STXBP1*-RD in other countries could investigate the generalizability of our findings. Second, the children of participating parents were between 2 and 18 years old. This study provides insights into the experiences of parents of children with *STXBP1*-RD across a broad age range, representing the experiences of parents with younger children, as well as parents with older children. However, based on our results, it is difficult to determine whether certain experiences are more prevalent or relevant to parents of children in specific age groups. Parental experiences may vary significantly depending on their child’s life stage, for instance, because children’s needs and the challenges faced by parents may evolve over time. To better understand age-specific parental experiences, parental experiences can be studied in more detail in narrower age groups. Lastly, participants were interviewed individually or as a couple. During the interviews with couples, parents were allowed to complement and react on each other’s responses. For instance, partners provided relevant background information or examples to each other’s responses, which potentially enriched the data. However, a potential risk is that some parents may have been less forthcoming with their partners being present. The interviewer endeavored to create a safe environment where parents felt comfortable to respond honestly to questions.

## Conclusions

Our analysis of the experiences of parents of children with *STXBP1*-RD contributes to a better understanding of how the child’s disorder and the level of care required impact family life. Our verbs-based framework highlights that parents are constantly occupied with (1) *caring* for their child to secure its safety and to care for all the child’s basic and medical needs. Parents are (2) *recognizing* and verbalizing their child’s needs to make them intelligible for their social environments. In addition to direct child-related responsibilities, parents are tasked with (3) *searching* for and arranging suitable care and support to obtain necessary care. Moreover, parents are (4) *balancing* childcare with other demands on their families, and (5) *coping* with emotional impacts. In sum, living with a child with *STXBP1*-RD is hard work. But it is not *only* hard work: parents also emphasize their children’s wellbeing and positive characteristics, and the valuable lessons they had learned from living with their child with *STXBP1*-RD. Our results show the need for a broad approach to support *STXBP1* patient families, encompassing not only the development of new medical treatments, but also improving other therapies and providing social support for the entire family, including providing emotional support through social workers, assistance with administrative tasks via ‘case-managers’, and improving information provision during the diagnostic process by healthcare professionals. The *verbs*-based framework developed in this study might also be useful to other research groups tasked with understanding disease burden in children with neurodevelopmental disorders and their families, and in future priority setting for research and care, not only in *STXBP1*-RD but also in other neurodevelopmental disorders.

## Electronic supplementary material

Below is the link to the electronic supplementary material.

Appendix 1: Interview guide BRAINmodel project

Appendix 2: Characteristics of respondents and their children

## Data Availability

The datasets generated and/or analyzed during the current study are not publicly available because the respondents agreed to be interviewed on the condition that the interview transcripts would be kept confidential.
