# Supplementary material for: ‘You constantly have to be switched on’: A qualitative interview study of parents of children with STXBP1-related disorders in the Netherlands
Source: Orphanet J Rare Dis. 2025 Feb 27;20:89. doi: 10.1186/s13023-024-03314-7 (PMC11869610; doi:10.1186/s13023-024-03314-7)
Supplement: Supplementary file 1 — Appendix 1: Interview guide BRAINmodel project [file 13023_2024_3314_MOESM1_ESM.docx]

**Appendix 1: Interview guide BRAINmodel project**

*For ethical research on the experiences and perspectives of parents of children with monogenetic neurodevelopmental disorders (mNDD)*

**Diagnose**

- *Can you describe what you noticed about your child after birth / in the first years of life?*
- *How was your child diagnosed?*
  - *How long did that take?*
  - *Which doctors were involved?*
  - *Have other diagnoses been considered?*
  - *How did you experienced the diagnostic process (from the first symptoms to getting the diagnosis)?*
- *What does this diagnosis mean to you?*
- *What does this diagnosis mean for your child? For example, in the care that your child receives? Are there any benefits for your child from having this diagnosis confirmed? Are there any disadvantages?*
- *What do you expect - based on this diagnosis - from the future development of the condition in your child?*

**Care**

- *What symptoms does your child currently have?*
- *What symptoms does your child suffers from most?*
- *Which of your child's symptoms are the most distressing for you (and your family)? (optional: can you describe the impact of seizures/behaviour problems /communication problems on the family life?)*
- *How is the care system for your child organised?*
  - *Where, from whom, and when does your child receive which kind of care?*
  - *Which doctor do you have the most contact with? Is that also the primary care provider?*
  - *What are your experiences on arranging appropriate care? Positive/negative experiences?*
  - *To what extent would you describe received care as consistent? (For example when switching schools, or changes in healthcare regulations?)*
- *Do you think the care and the organization of the care meet the needs of you and your child? What's going well? What are barriers?*
- *What are the most important (unmet) care needs for your child and for yourself?*

**Medical treatment**

- *Is your child currently receiving drug treatment(s)? If yes, which one?*
  - *What is the (treatment) goal?*
  - *To what extent does your child benefit from these treatments?*
  - *To what extent does your child suffer from side effects of these treatments?*
- *What should be the target of new (drug) treatments?*
- *To what extent are you concerned with possible (new) treatments that can reduce your child's symptoms? Why?*

**Emotional impact**

- *How does caring for your child affect daily life?*
  - *Of parents (and their relationship)*
  - *Of siblings*
  - *In relationships with others (e.g., family/friends/school)*
  - *Financial situation*
- *In which domain does the most important (mental) impact lie?*
- *To what extent can you also talk about a positive impact? Can you explain that?*

**Perspectives on tissue donation for research projects**

- *You have been given an explanation of a new technology with which human cells (such as skin cells) can develop into neuronal cell models in the laboratory. Can you tell us how you see this technology? What are your initial thoughts on the use of these neuronal cell models in research and treatment development?*
- *When you think of your child's stem cells being reprogrammed into brain cells in the lab, do those cells have a special meaning for you / a special relationship with your child? Are such neuronal cell models of a different significance to you than, for example, cell models that consist of other types of tissue (eg blood cells, intestinal cells, heart cells)? Can you explain why?*
- *Would you be willing to donate stem cells from your child for the development of neuronal cell models? What is your main motivation/goal in this?*
- *Are there any drawbacks to donating your child's cells for research? If so, which are they?*
- *What would you like to know/What information would you like to have in order to make an informed choice about whether or not donating stem cells for neuronal cell models?*

**Regulation neuronal cell models**

- *Would you like to be informed about what is happening with your child's neuronal cell models? What would be the right way to give this information to you, and when?*
- *Would you like to participate in decision-making on the (future) use of these cells? Why? And in what way?*
- *What are your perspectives on the accessibility to neuronal cell models; who should be allowed to use this tissue, and for what? (The doctors/researchers, researchers at other scientific institutions, researchers abroad, pharmaceutical or biotech companies?)*
- *Are there any limits to the application of neuronal cell models for you? What are these limits?*

**Perspective treatment options**

- *Neuronal cell models made from stem cells of a child with NDD can be used to see whether (existing) drugs 'work' in the brain cells and thus may have a positive effect on symptoms in the child. This testing of medicines on patient's own cells in the laboratory is called 'drug screening'. Suppose that from the screening there appears to be a suitable treatment that could possibly reduce the symptoms of your child. Would you be willing to have this treatment tested on your child?*
  - *Why/why not?*
  - *Under what conditions?*
- *To what extent do you think this new approach can contribute to the current standard of care for children with (monogenetic) NDD?*
- *What do you expect from the treatment process and the effects of the treatments?*
  - *Do you see disadvantages in selecting treatments in this way?*
- *What symptom(s) would you like to be prioritized in new therapies?*
- *What are your perspectives on the accessibility of this therapy?*
  - *Which patients should be eligible for treatment based on this approach?*
  - *What are your perspectives on the funding of these treatments? What do you find important in this? To what extent are you concerned about the funding of new treatments?*

**Information provision** (if time left)

- *Does the ‘rarity’ of the condition mean anything to you? In a practical sense? In the way you experience your child's diagnosis?*
- *Where (from which sources) do you get the most important information about your child's condition and care?*
- *To what extent do you think there is sufficient knowledge about the condition?*
  - *In your social environment*
  - *At healthcare providers*
- *Do you have contact with parents with children with NDDs based on the same mutations/similar mutations? What does this contact mean for you?*

**Respondent demographics**

- *Parent/caregiver/other role*
- *Man/Woman*
- *Age*
- *Educational background*
- *Ethnic background*
- *Age child*
- *Genetic mutation child*
- *Age of diagnosis*
