# Supplementary material for: ‘You constantly have to be switched on’: A qualitative interview study of parents of children with STXBP1-related disorders in the Netherlands
Source: Orphanet J Rare Dis. 2025 Feb 27;20:89. doi: 10.1186/s13023-024-03314-7 (PMC11869610; doi:10.1186/s13023-024-03314-7)
Supplement: Supplementary file 2 — Appendix 2: Characteristics of respondents and their children [file 13023_2024_3314_MOESM2_ESM.docx]

**Appendix 2: Characteristics of respondents and their children.**

| Sample characteristics | Results |
| --- | --- |
| Included respondents  Mother  Father  Mother and father  Relationship status  In a relationship/married  Not in a relationship/married  Employment status  Full-time job  Part-time job  Not employed (temporarily/permanently) | 9  2  5    13  3    5  12  4 |
| Age of child  2-6 years  7-11 years  12-18 years | 5  7  4 |
| Sex of child  Female  Male | 6  10 |
| Clinical characteristics of child  Cognitive impairment  Verbal communication  Verbal/some words  Nonverbal  Ability to walk  Ability to walk without assistance  Wheelchair dependent  Epilepsy  No seizures  Seizures  Past seizures  Current seizures | 15  5  11  12    4  4  12  6  6 |
